# Stress perception and associated factors among patients with Parkinson’s disease: a cross-sectional study after the COVID-19 pandemic

**DOI:** 10.1186/s12888-024-05972-0

**Published:** 2024-07-23

**Authors:** Yanhong Pan, Dandan Liang, Lingjie Lu, Zishan Yu, Bo Wang, Wei Luo, Ping Wang, Sheng Wu

**Affiliations:** 1 Nursing Department, The Second Affiliated Hospital of Zhejiang University School of Medicine, Zhejiang Province, China; 2 Department of Neurology, The Second Affiliated Hospital of Zhejiang University School of Medicine, Binjiang Campus, Zhejiang Province, China; 3 Department of Rehabilitation, The Second Affiliated Hospital of Zhejiang University School of Medicine, Binjiang Campus, Zhejiang Province, China

**Keywords:** Parkinson’s disease, Stress perception, Anxiety, COVID-19 pandemic

## Abstract

**Background:**

Parkinson’s disease (PD) is a serious neurodegenerative disease that brings great stress to the physical and mental health of patients. At the same time, long-term treatment will also bring great economic losses and social burden to the family and society, especially after COVID-19 pandemic. The aim of this study is to analyze the current status of stress perception and anxiety in patients with PD and explore the influencing factors after the COVID-19 pandemic.

**Methods:**

This study used the convenient sampling method to select the research objects of patients with PD who were outpatients or inpatients in a general public hospital in Hangzhou, Zhejiang Province, and the survey time was from February 2023 to March 2023. The measurements included the General information questionnaire, The Perceived Stress Scale (PSS) and The Self Rating Anxiety Scale (SAS). SPSS 21.0 software was used for data statistical analysis.

**Result:**

394 out of 420 patients with PD completed the questionnaire. The stress perception score of PD was (16.41 ± 6.435) and the anxiety score was (54.77 ± 10.477). The stress perception scores of patients with PD were significantly different in gender, age, educational, occupation, nature of costs, time of sleep, quality of sleep, duration of disease, way of medical treatment and anxiety level (*p* < 0.05). Among them, age, duration of disease, public expenses, online remote therapy and anxiety level were the main influencing factors of stress perception in patients with PD (*p* < 0.05). Besides, there were significant differences in gender, educational, nature of costs, time of sleep, quality of sleep and duration of disease in anxiety among patients with PD (*p* < 0.05).

**Conclusion:**

After the COVID-19 pandemic, the level of stress perception and anxiety in patients with PD is high, and the influencing factors are complex.

## Background

PD is the second common neurological degenerative disorder after Alzheimer’s disease. Its clinical manifestations include motor symptoms such as bradykinesia, rest tremor, and rigidity, as well as non motor symptoms such as constipation, mental disorders, sleep disorders, and autonomic dysfunction [1]. It is characterized by profound loss of substantia nigra cells in the pars compacta and accumulation of aggregated α-synuclein in specific brainstem, spinal cord, and cortical regions [2]. With the advent of aging society in China, the incidence of PD will increase year by year. It is expected that by 2030, the number of PD cases in China will reach nearly 5 million, accounting for nearly half of the world’s PD patients [3]. The symptoms of patients with PD often become more severe with the increase of the duration of the disease, which greatly affects the quality of life of patients and increases the economic burden [4]. In addition, due to the symptoms of dysfunction, movement disorder, cardiopulmonary dysfunction, speech dysfunction, cognitive dysfunction and mental dysfunction [5], patients with PD will lead to the decline of their self-management ability [6], the increase of fear of falling [7], stigma, anxiety and depression and other negative health emotions will also appear [8–11]. Especially after the COVID-19 pandemic, the motor and non-motor symptoms of PD patients have been directly or indirectly affected to some extent, such as bradykinesia, stiffness, rigidity and gait instability have worsened, and anxiety, depression, fatigue, pain, insomnia and cognitive impairment have also been aggravated [12–14]. In addition, for PD patients with older age, advanced disease duration, reduced medication use, or complications, the progression of disease may be accelerated, and the mortality will also increase, bringing great physiological and psychological stress to patients [15].

Stress perception is defined as the individual’s cognitive evaluation of situations and events beyond their own ability, through the dynamic cognitive evaluation process, the individual’s own physiological and psychological reactions [16]. Its operational definition can be defined as the individual’s unpleasant psychological and emotional feelings under the conditions of interacting with the external environment and beyond their own control, the most common experiences included frustration, tension, and anxiety [17]. The stress vulnerability hypothesis theory [18, 19] suggests that positive factors originally have a buffering function, but will be lost after the risk reaches a certain level. That is, for individuals with positive qualities, good adaptability is based on the premise of less stressful events, if the stressful events are too large, it will significantly weaken the individual’s ability to adapt to the environment. Studies have shown that high levels of stress perception can increase people’s anxiety and depression [20], affect their sleep quality and physical and mental health [21], and positively predict suicidal behavior in patients with depression [22]. Meanwhile, there is a significant correlation between stress perception and people’s quality of life, violence, self-efficacy, subjective well-being, risk perception, and mental health. At present, the research on stress perception mainly focuses on students, pregnant women, teachers, medical staff, etc. [23–27]. , while research on stress perception in PD patients has not yet been found.

In summary, this study mainly analyzed the current status of stress perception and anxiety in patients with PD after the COVID-19 pandemic, and explored its influencing factors, so as to provide a theoretical basis for formulating intervention programs that can effectively improve the level of stress perception and adverse health emotions in patients with PD in the future.

## Materials and methods

### Designs and participants

This cross-sectional survey used the convenient sampling method to select the research objects of PD patients who were outpatients or inpatients in a general public hospital in Hangzhou, Zhejiang Province, and the survey time was from February 2023 to March 2023. Inclusion criteria: (1) Age ≥ 18 years old; (2) Patients diagnosed with PD according to the clinical diagnostic criteria of the Movement Disorder Society (MDS) [28]; (3) Informed consent and voluntary participation in this study; (4) Be conscious and able to clearly understand the questionnaire content. Exclusion criteria: (1) Patients with major diseases (such as advanced cancer, organ failure, etc.) or obvious physiological defects; (2) History of mental illness; (3) Severe communication impairment or dementia; Unable to cooperate.

The sample size of measurement data was estimated by the formula *N* = 4*U*_*a*_^2^S^2^/*δ*^2^ [29]. According to the pre-survey of this study, the standard deviation of the dependent variable (stress perception score) was *S* = 6.13, and the allowable error *δ* was set as [0.25 S, 0.5 S] [30], *a* = 0.05, then *U*_*a*_=1.96. The sample size was calculated from 61 to 246 according to the formula, and the release was determined to be 20% considering the missing sample, so that the final sample size was 73 to 295. In order to ensure adequate sample size, 420 questionnaires were finally distributed and 420 were returned, of which 394 were valid questionnaires, with an effective rate of 93.8%.

### Data collection

Experts will conduct standardized training for all investigators, who will understand the contents of the questionnaire in detail, and explain the purpose and significance of the survey and the matters needing attention when filling out the questionnaire with unified guide, and a total of 420 questionnaires were distributed on the spot in the form of paper-pencil test. Finally, 394 valid questionnaires were obtained after removing invalid questionnaires, with effective recovery rate of 93.8%.

### Measurements

Data was collected using The general information questionnaire, Perceived Stress Scale (PSS) and Self-Rating Anxiety Scale (SAS). Except for the General information questionnaire, the other scales were mature scales at home and abroad.

The general information questionnaire was designed by the researchers after consulting the literature according to the purpose and content of the study. It included 11 variables: gender, age, educational, occupations, ethnicity, means of medical payment, comorbidity, time of sleep, quality of sleep, duration of illness, and means of transportation.

PSS was developed by Cohen [31] in 1983, and then translated and revised by Wang Zhen et al. [32]in 2015. It is used to evaluate the situation that individuals feel it is difficult to control, difficult to predict or overload in life. The scale consisted of 10 items, which were assigned 0 (never) to 4 (always) by Likert5 scoring method. Items 4, 5, 7 and 8 were reverse scoring, with a total score of 0–40. The higher the score of the study subject, the higher the level of stress perception. The Cronbach’s *a* coefficient of this study was 0.788.

SAS was a clinical measurement tool developed by Zung [33]in 1971 to assess the subjective symptoms of anxiety in patients. The scale consists of 20 items, with values ranging from 1 (none or very few) to 4 (most or all). Items 5, 9, 13, 17, and 19 were reverse scoring. The total rough score (X) was obtained by adding the scores of each item, and the total rough score (X) was multiplied by 1.25 to obtain the final score (Y). According to the results of the Chinese norm, the cut-off value of SAS is 50 points, with a score less than 50 points indicating normal anxiety, a score of 50–59 points indicating mild anxiety, and a score of 60–69 points indicating moderate anxiety; a score of 70 or above indicates severe anxiety. The Cronbach’s *a* coefficient of this study is 0.826.

### Statistical analysis

SPSS 21.0 software was used for data statistical analysis, the statistical analysis was performed by two-sided test, with *α* = 0.05 as the test level, and *p* < 0.05 as the difference being statistically significant.

1. Descriptive analysis: The mean (*M*), standard deviation (*SD*), frequency and composition ratio were used to describe the status of the general information, stress perception and anxiety in patients with PD;
2. Univariate analysis: *t*-test or analysis of variance was used to explore the differences of stress perception and anxiety in different demographic characteristics of PD patients;
3. Regression analysis: Multiple linear regression was used to analyze the effects of general demographic characteristics and anxiety on stress perception of patients with PD.

## Result

394 out of 420 PD patients completed the questionnaire. The stress perception score of PD patients is (16.41 ± 6.435), and the anxiety score is (54.77 ± 10.477), indicating an overall level of mild anxiety. The proportion of people with anxiety is relatively high (65.0%), and 32.0% of them had mild anxiety. As shown in Table 1.

**Table 1:** Study variable descriptions (*n* = 394)

Variables		Mean ± SD / *N* (%)
**Stress perception score**		16.41 ± 6.435
**Anxiety score**		54.77 ± 10.477
**Anxiety level**	Normal	138(35.0)
	Mild anxiety	126(32.0)
	Moderate Anxiety	95(24.1)
	Severe anxiety	35(8.9)

Among 394 patients, 201 were female and 193 were male; the average age was (59.44 ± 11.799), of which 60–79 years old accounted for the largest proportion of 51.5%; most of them had junior high school education or below (35.5%); and the occupations that were not easily classified were the most (28.9%); the Han ethnic group accounted for the largest proportion (93.9%); most of the means of medical payment were medical insurance (45.4%); the number of people with comorbidities was 227; most of the time of sleep was 5–7 h/d (60.4%) and the quality of sleep was general (54.0%); 151 (38.3%) had a disease duration of more than 10 years; most patients were treated by short-distance public transport (29.7%). As shown in Table 2.

The anxiety scores of patients with PD was significantly different in gender, educational, means of medical payment, time of sleep, quality of sleep and duration of disease (*p* < 0.05), but there was no significant difference in age, occupation, ethnicity, comorbidity and means of transportation (*p* > 0.05). PD patients with male, bachelor degree or above, public expense, duration of sleep ≥ 8 h/d and good sleep quality, duration of disease < 5 years had lower anxiety scores. As shown in Table 2.

**Table 2:** Anxiety scores of PD patients with different characteristics (*n* = 394)

Variables	*N* (%)	Score (Mean ± SD)	t /F	*P*
**Gender**				
Male	193(49.0)	53.65 ± 10.101	-2.076	0.039
Female	201(51.0)	55.84 ± 10.742		
**Age**				
≤ 40 years old	36(9.1)	50.92 ± 8.241	1.979	0.133
41–59 years old	142(36.1)	54.82 ± 9.204		
60–79 years old	203(51.5)	55.39 ± 11.177		
≥ 80 years old	13(3.3)	55.15 ± 15.726		
**Educational**				
Junior high school degreeor below	140(35.5)	57.23 ± 10.577	4.422	0.005
Senior high school	90(22.8)	53.99 ± 11.221		
Associate degree	79(20.1)	53.70 ± 9.493		
Bachelor degree or above	85(21.6)	52.53 ± 9.718		
**Occupations**				
Leaders of enterprise unit	52(13.2)	52.63 ± 9.911	1.958	0.060
Professional and technical personnel	94(23.9)	52.72 ± 9.831		
Production and transportation personnel	20(5.1)	58.90 ± 9.159		
Business and service personnel	38(9.6)	54.55 ± 11.519		
Agricultural, forestry, animal husbandry, fishery, water conservancy production personnel	44(11.2)	57.30 ± 12.262		
Staff and related personnel	28(7.1)	54.14 ± 8.864		
Military	4(1.0)	59.75 ± 24.918		
Other	114(28.9)	55.77 ± 9.735		
**Ethnicity**				
Han	370(93.9)	54.69 ± 10.554	1.697	0.150
Zhuang	5(1.3)	47.40 ± 5.941		
Hui	5(1.3)	57.40 ± 11.014		
Manthu	4(1.0)	52.00 ± 4.546		
Other	10(2.5)	61.10 ± 8.239		
**Means of medical payment**				
Self funded	124(31.5)	55.97 ± 11.061	3.069	0.028
Medical insurance	179(45.4)	54.80 ± 9.987		
Public expense	57(14.5)	51.16 ± 9.424		
Other	34(8.6)	56.24 ± 11.505		
**Comorbidity**				
No	167(42.4)	54.48 ± 10.445	-0.467	0.641
Yes	227(57.6)	54.98 ± 10.519		
**Time of sleep**				
≤ 4 h/d	90(22.8)	59.20 ± 11.060	11.163	< 0.001
5–7 h/d	238(60.4)	53.64 ± 9.537		
≥ 8 h/d	66(16.8)	52.79 ± 11.344		
**Quality of sleep**				
Well	70(17.8)	47.80 ± 7.829	37.411	< 0.001
General	213(54.0)	54.15 ± 9.201		
Poor	111(28.2)	60.34 ± 11.295		
**Duration of illness**				
< 5 years	110(27.9)	52.95 ± 9.848	3.250	0.040
5–10 years	133(33.8)	54.58 ± 11.027		
> 10 years	151(38.3)	56.26 ± 10.267		
**Means of transportation**				
Online remote medical treatment	26(6.6)	54.92 ± 8.328	2.446	0.46
Self driving or taxi	113(28.7)	53.20 ± 10.363		
Short distance public transport	117(29.7)	53.70 ± 10.020		
Long distance public transport	69(17.5)	56.67 ± 9.475		
Other	69(17.5)	57.17 ± 12.465		

Note: h/d: hour/day

The stress perception scores of patients with PD was significantly different in gender, age, educational, occupation, means of medical payment, time of sleep, quality of sleep, duration of disease, means of transportation and anxiety level (*p* < 0.05), but there was no significant difference in race and comorbidity (*p* > 0.05). PD patients with male, age ≤ 40 years old, bachelor degree or above, leaders of enterprise unit, public expense, time of sleep 4–7 h/d and good sleep quality, duration of disease < 5 years, short distance public transportation and normal anxiety level had lower stress perception scores. As shown in Table 3.

**Table 3:** Stress perception scores of PD patients with different characteristics (*n* = 394)

Variables	*N* (%)	Score (Mean ± SD)	t /F	*P*
**Gender**				
Male	193(49.0)	15.71 ± 6.410	-2.114	0.035
Female	201(51.0)	17.07 ± 6.405		
**Age**				
≤ 40 years old	36(9.1)	12.42 ± 6.308	5.724	0.001
41–59 years old	142(36.1)	16.61 ± 5.640		
60–79 years old	203(51.5)	16.82 ± 6.701		
≥ 80 years old	13(3.3)	18.77 ± 7.293		
**Educational**				
Junior high school degreeor below	140(35.5)	18.11 ± 5.536	5.934	0.001
Senior high school	90(22.8)	15.92 ± 7.294		
Associate degree	79(20.1)	15.77 ± 6.263		
Bachelor degree or above	85(21.6)	14.69 ± 6.464		
**Occupations**				
Leaders of enterprise unit	52(13.2)	14.17 ± 6.045	2.436	0.019
Professional and technical personnel	94(23.9)	15.39 ± 6.901		
Production and transportation personnel	20(5.1)	18.60 ± 4.465		
Business and service personnel	38(9.6)	16.68 ± 6.646		
Agricultural, forestry, animal husbandry, fishery, water conservancy production personnel	44(11.2)	18.34 ± 6.387		
Staff and related personnel	28(7.1)	16.43 ± 5.972		
Military	4(1.0)	14.50 ± 8.386		
Other	114(28.9)	17.10 ± 6.207		
**Ethnicity**				
Han	370(93.9)	16.38 ± 6.552	0.308	0.873
Zhuang	5(1.3)	14.40 ± 2.408		
Hui	5(1.3)	17.40 ± 3.782		
Manchu	4(1.0)	16.00 ± 3.162		
Other	10(2.5)	18.00 ± 5.518		
**Means of medical payment**				
Self funded	124(31.5)	17.24 ± 5.984	5.270	0.001
Medical insurance	179(45.4)	16.39 ± 6.327		
Public expense	57(14.5)	13.61 ± 6.646		
Other	34(8.6)	18.15 ± 7.042		
**Comorbidity**				
No	167(42.4)	16.51 ± 6.136	0.288	0.774
Yes	227(57.6)	16.33 ± 6.659		
**Time of sleep**				
≤ 4 h/d	90(22.8)	18.07 ± 6.425	3.994	0.019
5–7 h/d	238(60.4)	15.85 ± 6.192		
≥ 8 h/d	66(16.8)	16.14 ± 7.005		
**Quality of sleep**				
Well	70(17.8)	13.40 ± 6.619	15.805	< 0.001
General	213(54.0)	16.21 ± 6.033		
Poor	111(28.2)	18.68 ± 6.267		
**Duration of illness**				
< 5 years	110(27.9)	14.76 ± 6.449	6.272	0.002
5–10 years	133(33.8)	16.43 ± 6.420		
> 10 years	151(38.3)	17.58 ± 6.215		
**Means of transportation**				
Online remote medical treatment	26(6.6)	18.35 ± 4.732	3.125	0.015
Self driving or taxi	113(28.7)	15.98 ± 7.329		
Short distance public transport	117(29.7)	15.09 ± 5.948		
Long distance public transport	69(17.5)	17.90 ± 5.228		
Other	69(17.5)	17.10 ± 6.435		
**Anxiety level**				
Normal	138(35.0)	11.87 ± 5.808	72.448	< 0.001
Mild anxiety	126(32.0)	16.85 ± 5.001		
Moderate Anxiety	95(24.1)	19.65 ± 4.349		
Severe anxiety	35(8.9)	23.89 ± 5.217		

Note: h/d: hour/day

Based on the results of univariate analysis, statistically significant related factors were used as independent variables and stress perception as dependent variables. Multiple linear regression analysis was used to explore the influencing factors of stress perception in PD patients. Dummy variables were set for multiple categorical variable, as shown in Table 4. The results of multiple linear regression analysis show that the variance inflation factor of each variable is less than 10, indicating that there is no serious multicollinearity problem among the variables. At the same time, there were 10 variables entered into the regression equation, which could collectively explain 39.8% of the variation in stress perception in PD patients. Among them, age, duration of disease, public expenses, online remote therapy and anxiety level were the main influencing factors of stress perception in PD patients (*p* < 0.05). As shown in Table 5.

**Table 4:** Assignment method of independent variables

Independent variable	Assignment method
**Gender**	Male = 1; Female = 2
**Age**	≤ 40 years old = 1; 41–59 years old = 2; 60–79 years old = 3; ≥80 years old = 4
**Educational**	Junior high school degreeor below = 1; Senior high school = 2; Associate degree = 3; Bachelor degree or above = 4
**Occupations**	Leaders of enterprise unit = 1, 0, 0, 0, 0, 0, 0; Professional and technical personnel = 0, 1, 0, 0, 0, 0, 0; Production and transportation personnel = 0, 0, 1, 0, 0, 0, 0; Business and service personnel = 0, 0, 0, 1, 0, 0, 0; Agricultural, forestry, animal husbandry, fishery, water conservancy production personnel = 0, 0, 0, 0, 1, 0, 0; Staff and related personnel = 0, 0, 0, 0, 0, 1, 0; Military = 0, 0, 0, 0, 0, 0, 1; Other = 0, 0, 0, 0, 0, 0, 0
**means of medical payment**	Self funded = 1, 0, 0; Medical insurance = 0, 1, 0; Public expense = 0, 0, 1; Other = 0, 0, 0
**Time of sleep**	≤ 4 h/d = 1; 5–7 h/d = 2; ≥8 h/d = 3
**Quality of sleep**	Well = 1; General = 2; Poor = 3
**Duration of illness**	< 5 years = 1; 5–10 years = 2; >10 years = 3
**means of transportation**	Online remote medical treatment = 1, 0, 0, 0; Self driving or taxi = 0, 1, 0, 0; Short distance public transport = 0, 0, 1, 0; Long distance public transport = 0, 0, 0, 1; Other = 0, 0, 0, 0
**Anxiety**	Normal = 1; Mild anxiety = 2; Moderate Anxiety = 3; Severe anxiety = 4

Note: h/d: hour/day

**Table 5:** Multiple linear regression of factors of stress perception in PD patients

Variable	β	SE	t	*P*	VIF
**Constant**	3.709	2.449	1.514	0.131	—
**Age**	1.023	0.374	2.739	0.006	1,0.100
**Duration of illness**	0.788	0.318	2.478	0.014	1.041
**Means of medical payment**					
Public expense	-2.263	1.110	-2.038	0.042	2.410
**Means of transportation**					
Online remote medical treatment	2.684	1.182	2.272	0.024	1.359
**Anxiety**	3.423	0.288	11.863	< 0.000	1.238

Note: *R*^2^ = 0.419, Adjustment *R*^2^ = 0.398, *F* = 19.535, *P* < 0.000; *VIF*: Variance inflation factor

## Discussion

The purpose of this study is to investigate the current status of stress perception and anxiety in patients with PD after the COVID-19 pandemic and analyze the related influencing factors, so as to provide theoretical basis for developing intervention programs to improve the level of stress perception and anxiety in patients with PD in the future.

### Anxiety in patients with PD

The results showed that the anxiety score of PD patients was (54.77 ± 10.477), which was generally at a mild level of anxiety; the results of descriptive analysis showed that there was more people with anxiety (65.0%), and 32.0% of them had mild anxiety. The results of this study were consistent with Peng Shuangshuang’s [34] research, which showed that the incidence of anxiety in PD patients was 60%, of which mild anxiety accounted for 31.6%; Chai Bin et al. [35]showed that the incidence of anxiety in patients with PD was 53.3%; a meta-analysis by Broen et al. [11]showed that the incidence of anxiety in patients with PD was 31.0%. It can be seen that the incidence of anxiety in patients with PD is generally high, which may be due to anxiety as a psychological response to the stress of the disease, or it may be related to the neurochemical reaction of the disease itself, and anti-Parkinson’s disease drugs may also play a certain role in the pathogenesis of anxiety [10, 36]. In addition, during the COVID-19 pandemic, PD patients with or without COVID-19 experienced an increase in the severity of both motor and non-motor symptoms, including tremor, myotonia, mood changes, cognitive impairment, constipation, etc., which may be aggravate their anxiety level and the quality of life of patients [15].

The results of univariate analysis showed that the influencing factors of anxiety in patients with PD included age, educational, means of medical payment, time of sleep, quality of sleep and duration of disease. PD is an irreversible disease that progresses both motor and non-motor symptoms over time. Therefore, as the age and duration of the disease increases, the level of anxiety may also increase [37]. In addition, educational is also one of the reasons affecting the anxiety level of PD patients, which may be because thehigher the education level, the higher the cognitive level of patients, and the more ways they can acquire disease-related knowledge. They will have a more comprehensive understanding of the disease, have higher treatment compliance, and have relatively less fear of the disease, resulting in lower levels of anxiety [38]. The treatment of PD will bring great economic pressure, and the nature of the patient’s expenses determines how much they spend. Once they exceed their financial capacity, it will bring great pressure, which might lead to anxiety in some patients [3]. Quality of sleep has been considered as an important indicator of physical and mental health, and poor sleep quality will affect the psychological and emotional regulation ability of patients, thereby increasing their risk of anxiety and depression [39].

### Stress perception in patients with PD

The results of this study showed that the stress perception score of PD patients was (16.41 ± 6.435), which is higher than the research results of Zhang Xiang et al. [40]. , that is, the stress perception score of the general population is (12.59 ± 6.96). The core viewpoint of The Cognitive Phenomenon-Transactional model [41] points out that the changes of individual mental state and external stimulus scenarios jointly determine the individual’s cognitive evaluation of stressors. Compared with the general population, PD patients have changes in motor symptoms such as bradykinesia, rest tremor and myotonia, and non-motor symptoms such as constipation, mental disorders, sleep disorders and autonomic dysfunction [42]. Especially after the COVID-19 pandemic, these symptoms may be more or less worsen, seriously affecting their quality of life and subjective well-being. In addition, as a chronic disease, patients with PD need to take a variety of drugs multiple times a day for a long time to control and alleviate the disease, however, it generally has problems such as poor medication compliance [43], low self-management ability and self-efficacy [6, 44], which lead to ineffective treatment of the disease, thus forming a vicious circle and increasing the level of stress perception.

The results of univariate analysis showed that the influencing factors of stress perception in patients with PD included gender, age, educational, occupation, means of medical payment, time of sleep, quality of sleep, duration of disease, means of transportation and anxiety level. Among them, age, duration of disease, public expenses, online remote therapy and anxiety level are independent risk factor. The reason may be that the older the age and the longer the course of the disease, the worse the development of the physical and mental health of the patients, and the more external stimuli they face, such as social, physiological and psychological, leading to a higher level of stress perception [37]. In addition, the treatment of PD is a long-term process, during which a large amount of costs will be incurred [4]. According to statistics, the per capita economic burden of PD patients accounts for 17.9% of family income and 48.0% of per capita annual income, respectively [45]. It is expected that the total economic burden of PD patients will exceed $79 billion by 2037 [4]. If the means of medical payment is public expense, there will not be much economic pressure. The online remote treatment can reduce patients’ travel anxiety and waiting time for offline treatment, increase treatment efficiency, and thus reduce their stress perception level. Finally, stress perception is an individual’s cognitive assessment of situations and events beyond their abilities, while anxiety is a common negative emotional experience that occurs in the process of cognitive assessment [17]. Therefore, the higher the anxiety level of patients with PD, the more negative emotional experience they perceive, and the higher their stress perception level will be.

This study still has certain limitations. Firstly, this study is a cross-sectional study without follow-up of PD patients, and it is unclear what changes will occur in their stress perception levels over time. In future studies, longitudinal studies should be conducted to further explore the influencing factors of stress perception in patients with PD, so as to provide evidence for formulating effective intervention programs in the future. Secondly, this study is a self-reported measurement, and patients have strong subjective awareness, which may produce a large bias. In the future, it may be possible to combine self-evaluation and peer evaluation methods to evaluate, or to find or develop objective evaluation indicators for measurement and analysis. Finally, due to limitations in manpower, material resources and time, this study only selected PD patients from hospital in Zhejiang Province, which may lead to measurement bias or reporting bias, so it may not represent the situation of the entire PD population or other countries. In the future research, we can expand the research scope according to the differences of economy, region and culture, select more sample sizes, and increase the representativeness of the study.

## Conclusion

After the COVID-19 pandemic, the level of stress perception and anxiety in patients with PD is high, and the influencing factors are complex. In the process of caring for patients, medical workers should take into account the potential adverse effects of excessive stress, assess the level of stress perception in a timely manner, and develop personalized intervention programs according to the possible stress - perhaps making changes in the way of medical treatment, quality of sleep, medical insurance policies, etc. In the future, more types of studies can be carried out to explore the related issues of stress perception in patients with PD, so as to provide effective programs for improving their quality of life.

## Data Availability

The datasets used and analyzed during the current study are available from the corresponding author upon reasonable request.

## Abbreviations

PD: Parkinson’s Disease
SAS: The Self Rating Anxiety Scale
PSS: The Perceived Stress Scale
